# Using Intra-Aortic Balloon Pump for Management of Cardiogenic Shock Following Aluminum Phosphide Poisoning; Report of 3 Cases

**Published:** 2018-01-12

**Authors:** Ahmad Bagheri-Moghaddam, Hasan Abbaspour, Shahrad Tajoddini, Vahideh Mohammadzadeh, Ali Moinipour, Bita Dadpour

**Affiliations:** 1 Department of Anesthesiology, Faculty of Medicine, Mashhad University of Medical Sciences, Mashhad, Iran.; 2 Neuroscience Research Center, Institute of Neuropharmacology, Kerman University of Medical Sciences, Kerman, Iran.; 3 School of Pharmacy, Mashhad University of Medical Sciences, Mashhad, Iran.; 4 Department of cardiac surgery, Faculty of Medicine, Mashhad University of Medical Sciences, Mashhad, Iran.; 5 Medical Toxicology Research Center, School of Medicine, Mashhad University of Medical Sciences, Mashhad, Iran.

**Keywords:** Poisoning; shock, cardiogenic, intra-aortic balloon pumping, patient outcome assessment

## Abstract

**Introduction:**

Aluminium phosphide (Alp) poisoning mortality rate has been reported as high as 70-100%, and refractory hypotension and cardiogenic shock are the two most common presentations leading to death. Due to lack of specific antidote, all treatments are focused on supportive care and recently, intra-aortic balloon pump (IABP) has been suggested to treat cardiogenic shock resulting from toxic myocarditis. In the current paper, we introduce three Alp poisoned patients for whom IABP was applied to manage their refractory shock.

**Case presentation:**

Two men and one woman who were admitted to emergency department (ED) of Imam Reza academic Hospital, Mashhad, Iran due to intentional Alp poisoning are reported. The cases visited the ED shortly after ingestion and nearly all of them showed hypotension, tachycardia and metabolic acidosis during early hospitalization. Due to persistent shock state, despite receiving intravenous fluid therapy and vasopressor agents, IABP insertion was performed in these cases. Finally, one of them survived and the other two died.

**Conclusion:**

It still cannot be decided whether IABP insertion is effective in cases of Alp poisoning or not. It might be reasonable to try this intervention along with other conservative treatments in patients who survive more than 12 hours and consistently suffer from refractory hypotension.

## Introduction:

Aluminium phosphide (Alp), the main component of “Rice Tablet”, is frequently used as a rodenticide, fumigant and pesticide (1-5). It is easily available in some countries such as Iran and India, and as a result many cases of suicide attempts have been reported with Alp in these countries (5, 6). The exact mechanism of toxicity is not clear, but release of phosphine gas and subsequent cellular events including cytochrome oxidase inhibition and oxidative stress are proposed as the possible mechanism (7, 8). Mortality rate of Alp poisoning has been reported as high as 70-100%, and refractory hypotension and cardiogenic shock are the two most common presentations leading to death (9, 10). Due to lack of specific antidote, all treatments are focused on supportive care and researches are still in progress to make advancements in Alp poisoning management (11-15). Recently, intra-aortic balloon pump (IABP) has been suggested to treat cardiogenic shock resulting from toxic myocarditis following various poisonings (16, 17). There are some reports of IABP insertion regarding Alp poisoning, some successful and some not (18, 19). In the current paper, we introduce three Alp poisoned patients for whom IABP was applied to manage their refractory shock.

## Case presentation:

***Case 1***

A 20-year-old man who ingested Alp (5 grams of 56% powder) was admitted to ED with nausea, vomiting, diarrhea and abdominal pain. He was confused on admission. Vital signs were as follows: blood pressure (BP) =75/45 mmHg; respiratory rate (RR) = 35/minute; pulse rate (PR) = 100/minute; and oxygen saturation (SpO2) = 93% (in room air). Intravenous (IV) fluid therapy by normal saline 0.9% in addition to dopamine infusion was initiated. Six hours later, BP and SpO2 had dropped to 70/pulse and 90%, respectively, and PR had increased to 110/minute. Arterial blood gas (ABG) assessment revealed pH=6.9 and Hco3=8 mEq/L. Four vials of sodium bicarbonate were infused. Twelve hours later, he was admitted to intensive care unit (ICU) due to persistent hypotension and hypoxemia, as well as ventricular systoles, atrial fibrillation (AF) and left bundle branch block (LBBB) in electrocardiogram (ECG). On admission to ICU, vital signs were PR=120/minute, BP=79/45 mmHg and SpO2=88%. Fourteen hours after ingestion, he was intubated because of persistent shock state despite IV fluid therapy and administration of norepinephrine 20 micrograms/minute in addition to dopamine 5 to 15 microgram/kg/min. Thereafter, 0.5 mg digoxin was injected due to AF rhythm and low BP. Dilation of left ventricle and ejection fraction (EF) less than 25% was reported in echocardiography. IABP was inserted 18 hours after ingestion and after 97 hours on the 5^th^ day of admission, it was removed as BP and EF had improved. In echocardiography, which was performed on the 9^th^ day, EF was normal, but pulmonary arterial pressure (PAP) was 75 mmHg and right ventricle was dilated, and following cardiologist consultation, sildenafil and milrinone were initiated. Echocardiography revealed normal values on day 14. He was discharged with normal vital signs and laboratory test results.

***Case 2***

A 30-year-old woman was admitted to ED 2 hours after ingestion of half an Alp tablet. Vital signs on admission included: BP=95/70 mmHg, RR=14/minute, PR=77/minute, and SpO2=97% (in room air). She was admitted to ICU 6 hours later due to refractory hypotension despite administration of IV fluid therapy and vasopressor agents. On admission to ICU pulse rate was 114/minute and SBP/DBP was 80/50 mmHg. ABG revealed: SpO2=93%, pH=7.28, pCO2=16.5, HCO3=7.8 mEq/L and ScVO2=45%. She received IV fluid therapy, norepinephrine and dopamine, digoxin 0.5 mg, NAC (9 grams within 1 hour, 3 grams within 4 hours and 4 grams within 16 hours), vitamin C 1 gram/day, Calcium gluconate 1 gram every 8 hours. Finding of echocardiography was LVEF=30 -35%. She was intubated 12 hours after ingestion due to respiratory distress and SpO2 less than 88% despite oxygen therapy by reserve bag. IABP was inserted 11 hours later. After 24 hours, she developed ventricular arrhythmia and amiodarone and lidocaine were administered. After 40 hours and despite correction of acidosis, she suddenly developed cardiac arrest and after two times of CPR, was announced dead.

***Case 3***

A 17-year-old man was admitted to ED due to consumption of one Alp tablet 2.5 hours before admission. Vital signs were: RR=1 /min, O2sat=95% (in room air), PR=77/min and BP=65/pulse mmHg on admission. Seven hours later he was transferred to ICU. On admission to ICU blood pressure was not detectable, PR=120/min and SpO2=80%. ABG revealed pH=7.18, pCO2=44.7, HCO3=16.8 mEq/L and ScVO2=50%. He received IV fluid therapy in addition to infusion of norepinephrine and dopamine, digoxin 0.5 mg IV, vitamin E 100mg/daily, Vitamin C 1 gram/daily, dexamethasone and NAC (9 gram in one hour, 4 grams in 4 hours and 6 grams in 16 hours). Hypotension was refractory to hydration and infusion of high dose vasopressors. ECG revealed invert T in V3 to V6 leads and echocardiography showed global hypokinesia and LVEF=10-15%. IABP was inserted 4 hours after admission and in the next hour he was intubated. Systolic blood pressure was below 6 mmHg despite correction of acidosis and other supportive cares. Twenty hours after ingestion he developed sudden cardiopulmonary arrest and after 45 minutes of CPR he died. 

## Discussion:

Lethal dose of Alp is about 500 mg of a tablet (about a sixth of a tablet). Some factors might influence prognosis of patients for example length of exposure of tablet to air, whether it is solved in water or not and length of exposure to water; all these factors affect the rate of phosphine gas release before it is consumed. The most important complications of Alp poisoning are cardiovascular problems and in particular toxic myocarditis that might lead to severe heart dysfunction. However, cardiac function may be restored if the patient survives (20). 

It was reported that use of IABP could be successful in some cases of acute poisoning with severe cardiac dysfunction (21, 22). It seems that, for the first time, IABP was inserted in a 25 year old woman with severe toxicity of Alp (consumption of 10 tablets) in 2008, which was not successful (22). Siddaiah et al. reported the first successful use of IABP in a 22-year-old woman with cardiogenic shock following Alp consumption (16). Subsequently, the second successful use was reported by Mehrpour et al. in a 24-year-old woman in 2013 (7).

Annually, about 300 cases of Alp poisoning are referred to Imam Reza hospital, to which these 3 cases were admitted, and most of the cases are admitted to ICU. Due to the high rate of mortality in this type of poisoning and also a documented antidote not being available while there are some reports of successful treatment with IABP, we decided to apply IABP for our cases in addition to other supportive cares. We used IABP in three cases and it was successful in one case and he was discharged from hospital without any complication; yet we did not have success in the other two cases and both died. All cases consumed Alp intentionally. 

More investigations are still needed to decide if IABP insertion is effective or not in cases of Alp poisoning. However, the method of selecting appropriate cases is a matter of debate and needs to be investigated more.

## Conclusion:

It still cannot be decided whether IABP insertion is effective in cases of Alp poisoning or not. It might be reasonable to try this intervention along with other conservative treatments in patients who survive more than 12 hours and consistently suffer from refractory hypotension.

## References

[1] Mehrpour O (2012). Comment on “An update on toxicology of aluminum phosphide”. Daru.

[2] Mehrpour O, Keyler D, Shadnia S (2009). Comment on Aluminum and zinc phosphide poisoning. Clinical toxicology.

[3] Mehrpour O, Jafarzadeh M, Abdollahi M (2012). A systematic review of aluminium phosphide poisoning. Archives of Industrial Hygiene and Toxicology.

[4] Shadnia S, Mehrpour O, Soltaninejad K (2011). A simplified acute physiology score in the prediction of acute aluminum phosphide poisoning outcome. Indian J Med Sci.

[5] Shadnia S, Mehrpour O, Abdollahi M (2008). Unintentional poisoning by phosphine released from aluminum phosphide. Human & experimental toxicology.

[6] Mehrpour O, Singh S (2010). Rice tablet poisoning: a major concern in Iranian population. Human & experimental toxicology.

[7] Mehrpour O, Amouzeshi A, Dadpour B, Oghabian Z, Zamani N, Amini S (2014). Successful treatment of cardiogenic shock with an intraaortic balloon pump following aluminium phosphide poisoning. Archives of Industrial Hygiene and Toxicology.

[8] Hashemi-Domeneh B, Zamani N, Hassanian-Moghaddam H, Rahimi M, Shadnia S, Erfantalab P (2016). A review of aluminium phosphide poisoning and a flowchart to treat it. Arhiv za higijenu rada i toksikologiju.

[9] Kumar U, AG VK (2017). Fatal Aluminium Phosphide Poisonings in Nagamangala Taluq-A Retrospective Study. Indian Journal of Forensic Medicine & Toxicology.

[10] Agrawal VK, Bansal A, Singh RK, Kumawat BL, Mahajan P (2015). Aluminum phosphide poisoning: Possible role of supportive measures in the absence of specific antidote. Indian Journal of Critical Care Medicine : Peer-reviewed, Official Publication of Indian Society of Critical Care Medicine.

[11] Mehra A, Sharma N (2016). ECMO: A ray of hope for young suicide victims with acute aluminum phosphide poisoning and shock. Indian heart journal.

[12] Neki N, Shergill GS, Singh A, Kaur A, Nizami S, Singh T (2017). Recent Advances in Management of Aluminium Phosphide Poisoning. Int J Curr Res Med Sci.

[13] Mirakbari S (2015). Hot charcoal vomitus in aluminum phosphide poisoning-A case report of internal thermal reaction in aluminum phosphide poisoning and review of literature. Indian journal of anaesthesia.

[14] Taghaddosinejad F, Farzaneh E, Ghazanfari-Nasrabad M, Eizadi-Mood N, Hajihosseini M, Mehrpour O (2016). The effect of N-acetyl cysteine (NAC) on aluminum phosphide poisoning inducing cardiovascular toxicity: a case-control study. SpringerPlus.

[15] Halvaei Z, Tehrani H, Soltaninejad K, Abdollahi M, Shadnia S (2017). Vitamin E as a novel therapy in the treatment of acute aluminum phosphide poisoning. Turkish journal of medical sciences.

[16] Siddaiah LM, Adhyapak SM, Jaydev SM, Shetty GG, Varghese K, Patil CB (2009). Intra-aortic balloon pump in toxic myocarditis due to aluminum phosphide poisoning. Journal of medical toxicology.

[17] D’sa SR, Peter JV, Chacko B, Pichamuthu K, Sathyendra S (2016). Intra-aortic balloon pump (IABP) rescue therapy for refractory cardiogenic shock due to scorpion sting envenomation. Clinical Toxicology.

[18] Mehrpour O, Gurjar M (2017). Cardiogenic Shock: The Main Cause of Mortality in Acute Aluminum Phosphide Poisoning. Indian journal of critical care medicine: peer-reviewed, official publication of Indian Society of Critical Care Medicine.

[19] Bansal P, Giri S, Bansal R, Tomar LR (2017). Survival in a case of aluminum phosphide poisoning with severe myocardial toxicity. Indian Journal of Health Sciences and Biomedical Research (KLEU).

[20] Akkaoui M, Achour S, Abidi K, Himdi B, Madani A, Zeggwagh AA (2007). Reversible myocardial injury associated with aluminum phosphide poisoning. Clinical toxicology.

[21] Lane AS, Woodward AC, Goldman MR (1987). Massive propranolol overdose poorly responsive to pharmacologic therapy: use of the intra-aortic balloon pump. Annals of emergency medicine.

[22] Chacko J, Shivaprasad C (2008). Fatal aluminium phosphide poisoning due to myocardial depression refractory to high dose inotropic support and intra-aortic balloon counterpulsation. Indian Journal of Critical Care Medicine.

